# Silencing of heat shock protein 90 (hsp90): Effect on development and infectivity of *Ichthyophthirius multifiliis*

**DOI:** 10.1186/s12917-023-03613-4

**Published:** 2023-03-18

**Authors:** Mona Saleh, Abdel-Azeem S. Abdel-Baki, Mohamed A. Dkhil, Mansour El-Matbouli, Saleh Al-Quraishy

**Affiliations:** 1 Clinical Division of Fish Medicine, University of Veterinary Medicine, Veterinärplatz 1, Vienna, 1210 Austria; 2 Zoology Department, Faculty of Science, Beni-Suef University, Beni-Suef, Egypt; 3 Department of Zoology and Entomology, Faculty of Science, Helwan University, Cairo, Egypt; 4 Scchool of Biotechnology, Badr University in Cairo (BUC), Badr City, Cairo Egypt; 5 Zoology Department, College of Science, King Saud University, Riyadh, Saudi Arabia

**Keywords:** Ciliates, Protozoa, Parasites, Knockdown, mRNA, Gene expression

## Abstract

**Background:**

Recently, an increasing number of ichthyophthiriasis outbreaks has been reported, leading to high economic losses in fisheries and aquaculture. Although several strategies, including chemotherapeutics and immunoprophylaxis, have been implemented to control the parasite, no effective method is available. Hence, it is crucial to discover novel drug targets and vaccine candidates against *Ichthyophthirius multifiliis*. For this reason, understanding the parasite stage biology, host–pathogen interactions, molecular factors, regulation of major aspects during the invasion, and signaling pathways of the parasite can promote further prospects for disease management. Unfortunately, functional studies have been hampered in this ciliate due to the lack of robust methods for efficient nucleic acid delivery and genetic manipulation. In the current study, we used antisense technology to investigate the effects of targeted gene knockdown on the development and infectivity of *I. multifiliis*. Antisense oligonucleotides (ASOs) and their gold nanoconjugates were used to silence the heat shock protein 90 (hsp90) of *I. multifiliis*. Parasite stages were monitored for motility and development. In addition, the ability of the treated parasites to infect fish and cause disease was evaluated.

**Results:**

We demonstrated that ASOs were rapidly internalized by *I. multifiliis* and distributed diffusely throughout the cytosol. Knocking down of *I. multifiliis* hsp90 dramatically limited the growth and development of the parasite. In vivo exposure of common carp (*Cyprinus carpio*) showed reduced infectivity of ASO-treated theronts compared with the control group. No mortalities were recorded in the fish groups exposed to theronts pre-treated with ASOs compared with the 100% mortality observed in the non-treated control fish.

**Conclusion:**

This study presents a gene regulation approach for investigating gene function in *I. multifiliis in vitro*. In addition, we provide genetic evidence for the crucial role of hsp90 in the growth and development of the parasite, suggesting hsp90 as a novel therapeutic target for successful disease management. Further, this study introduces a useful tool and provides a significant contribution to the assessing and understanding of gene function in *I. multifiliis*.

## Background

*Ichthyophthirius multifiliis* is a ciliated protozoan parasite, which is recognized as a causative agent of one of the most pathogenic diseases of wild and cultured freshwater fish [1]. *I. multifiliis* causes ichthyophthiriasis, usually known as “white spot disease” because of the appearance of macroscopically observable parasites as white spots on the epidermis of infected fish. Wide temperature tolerance and a broad host range of the parasite are responsible for high morbidity and mortality in both farmed freshwater fish and ornamental fish [2]. The life cycle of the parasite consists of several morphologically distinct stages: a free-swimming, highly motile, and infective theront that penetrates the epithelium of the skin and gills, where it feeds on the host tissues and transforms into a large trophont [3, 4]. The trophonts become visible as individual white spots, based on which the disease acquires its name. Vigorous trophont growth in the gills causes asphyxiation and death. The parasite leaves the host and replicates within a protective cyst in the aqueous environment, where it undergoes numerous divisions to produce the next generation of theronts. However, successful encystment and theront production are partly a function of trophont size [5, 6].

Antisense oligonucleotides (ASOs) are macromolecules that target mRNAs before these are translated into proteins. Thus, ASOs can interfere specifically with gene expression and inhibit the production of the corresponding protein [7]. Inhibition of the gene expression occurs by a variety of mechanisms, depending on the chemical makeup of the AO and the site of hybridization to target mRNAs, via the Watson–Crick base pairing [8]. Oligonucleotides that do not support RNase H activity can affect the gene expression via translation arrest or alternative splicing [9]. Over the past decade, ASOs have been considered as a potential approach to investigate gene function in protozoan parasites, e.g., *Plasmodium falciparum* [9–12] and *Leishmania* species [12–17].

Gold nanoparticles (AuNPs) have a high affinity for biomolecules and have frequently been used as non-viral vectors for DNA delivery [17–21]. AuNPs surface-functionalized with oligonucleotides exhibit a high transfection efficacy and have been used to control gene expression [21–24]. Oligonucleotide-AuNP conjugates (ASNPs) readily enter the cells and function as composite antisense nanoconjugates, outperforming molecular ASOs in terms of stability and gene silencing ability [22]. It has been reported that the oligonucleotide-modified nanoparticles exhibit binding constants for complementary nucleic acids more than unmodified oligonucleotide equivalents. They are less vulnerable to dissociation by nuclease activity, display higher than 99% cellular uptake, can present oligonucleotides at a higher effective concentration than general transfection agents, and are non-toxic to cells under the investigated conditions [22].

In a previous study, by means of the shotgun proteomic approach, numerous differentially regulated common carp proteins were identified post exposure to *I. multifiliis* [25, 26]. In addition, we were able to discover six *I. multifiliis* proteins, including the heat shock protein 90 (hsp90), from the skin mucus samples of the infected fish [27]. The identified proteins were found to function in motility, development, and growth, as analyzed using the NCBI database entries of *I. multifiliis* (*Taxonomy ID*: 5932) and using the Search Tool for the Retrieval of Interacting Genes/Proteins (STRING) analysis tool. Among the identified proteins, hsps play a role in housekeeping and regulation of protein quality control under normal conditions. In addition, these are essential for cell survival and death decisions under conditions of stress [28]. For example, Hsp90 is involved in cell cycle control and cellular stress response in the protozoan parasite *Leishmania donovani* [29]. Hsp90 inhibition is lethal to *Trichomonas* growth, indicating its critical function in the survival of the parasite [30]. Hsp90 was detected in the cilia of *Tetrahymena* species and the molecular interactions stimulated by hsp90 were implicated in shaping the cortical modelling [31]. Genes required for protein assembly, folding, and translocation, including hsp70 and hsp90, are differentially reguted among the lifecycle stages of *I. multifiliis* [32]. However, gene functional studies are scarce in *I. multifiliis* because of the lack of genetic methods for studying and validating gene function.

We hypothesize that hsp90 could be targeted within the parasite stages to negatively affect the parasite, interrupt its life cycle, inhibit the production of theronts, or render theronts non-infectious. We used the antisense approach to target and inhibit hsp90 in *I. multifiliis*.

This work provides an effective resource to enhance our understanding of gene function of *I. multifillis*. In addition, it establishes the basis for evaluating potential drug targets and vaccine candidates for the control of this devastating fish pathogen.

## Results

### Collection of parasite stages

Stages of *I. multifiliis* were considered as active (survival) or motionless (dead), as described previously [45]. Antisense oligonucleotides were designed to target specific mRNA sequences selected to inhibit the expression of hsp90 in *I. multifiliis.* The nanoconjugates were citrate-capped gold nanoparticles functionalized with thiol-modified ASOs. Factors affecting the delivery process, such as the concentration of AO, transfection reagent, and sampling time for monitoring gene knockdown, were assessed and optimized.

### Design, selection, synthesis and delivery of *Ichthyophthirius multifiliis*-specific antisense oligonucleotides

**Fig. 1:**
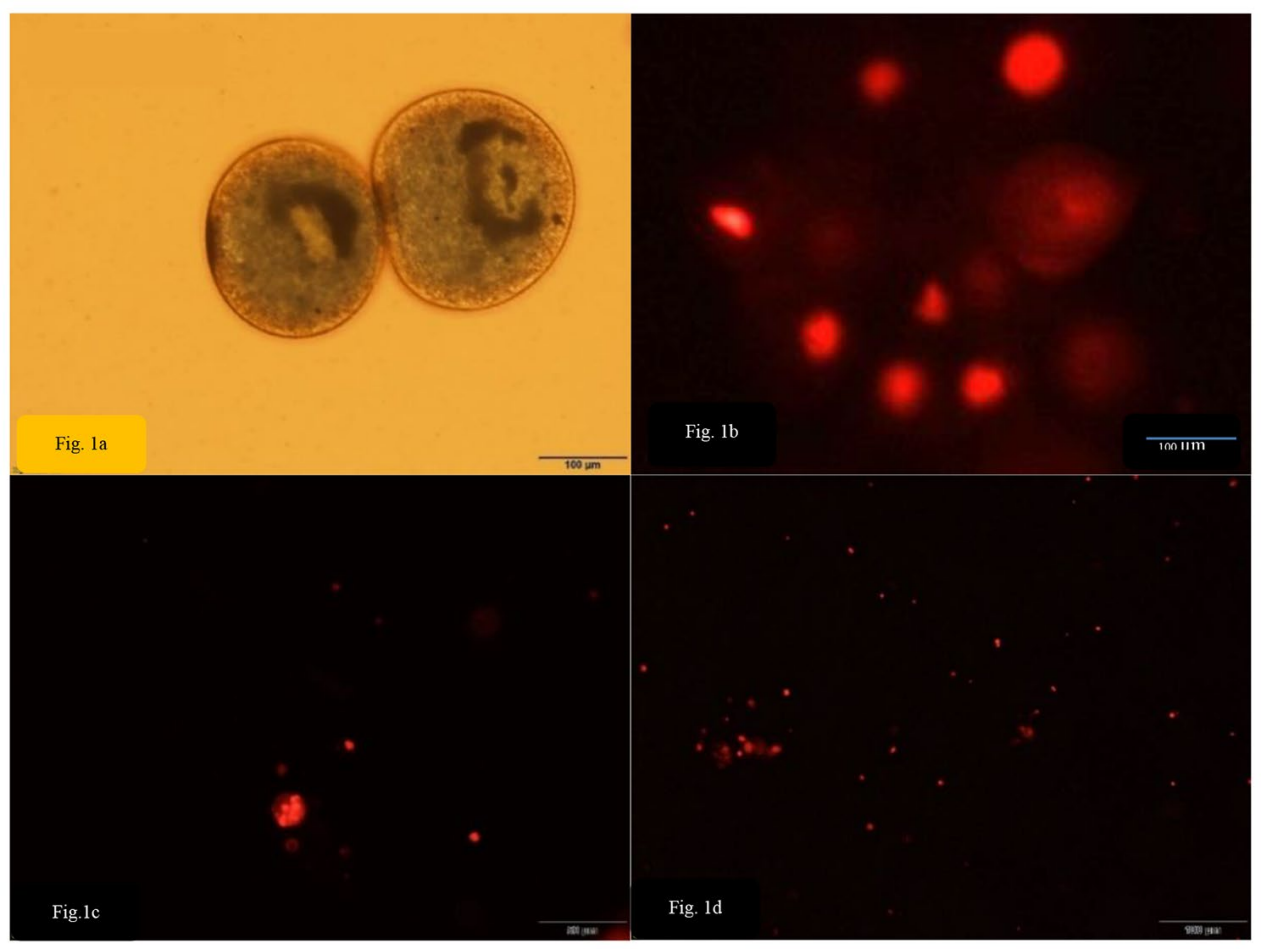
Developmental stages of *I. multifiliis*; Trophonts (a-b), tomocysts (c) and theronts (d). The figure (b-d) demonstrates the uptake of the cy5-labeled scrambled antisense (negative control)

The designed oligonucleotides were suited for both mRNA regulation and fluorescence microscopy by using different concentration of the AOSs (0,5 − 2 µM) and incubation periods from 10 min to 2 h. Viable *I. multifiliis* stages were collected (Fig. 1a-). Parasite stages were observed for successful transfection under a fluorescence microscope. After 2 h of incubation, successful introduction of fluorescently labeled oligonucleotides into *I. multifiliis* was seen (Fig. 1b-d). Observation of fluorescence in the nucleus and cytoplasm confirmed the particle uptake, with 99% of uptake observed at 2 µM. Practically every cell incorporated the ASOs. In addition, there were no differences in cellular morphology in untreated scrambled control cells. The observed fluorescence indicates that the oligonucleotides remain undigested by nucleases.

**Fig. 2:**
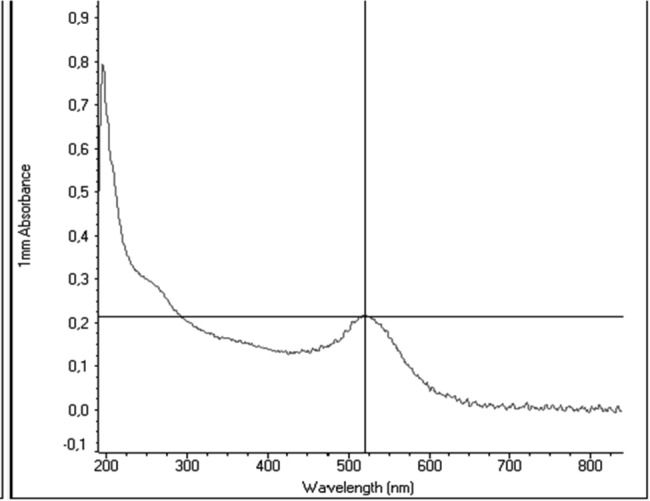
The figure demonstrates UV–vis analysis for gold nanoparticles with peak absorption at wavelength 523 nm

UV–Vis analysis of gold nanoparticles showed the maximum absorption at 523 nm, corresponding to the expected values for gold nanoparticles confirming the synthesizes of the AuNPs (Fig. 2).

### Characterization of nanoparticles

**Fig. 3:**
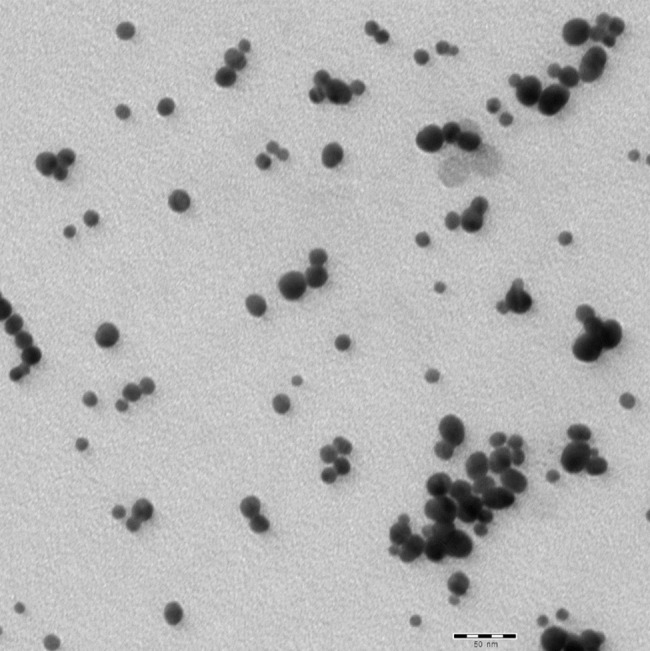
TEM micrograph shows spherical morphology with a mean size of 18 nm. (Scale bar = 50 nm)

TEM imaging revealed gold nanoparticles were spherical, with a mean diameters of 18 nm as shown in Fig. 3.

**Fig. 4:**
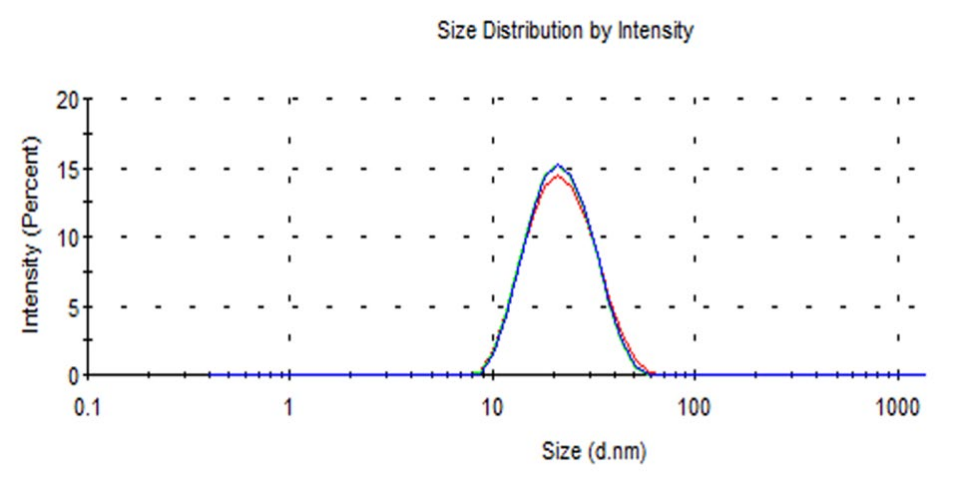
Particle size distribution with a narrow size distribution range showing one peak at 23 nm showing the homogenous distribution of the particles. All data were expressed as means ± SD (n = 3)

The Dynamic light scattering (DLS) measurements showed one peak at 23 nm for gold nanoparticles confirming the homogenous distribution of the synthesized gold nanoparticles (Fig. 4).

### Gene expression analysis by qRT-PCR

**Fig. 5:**
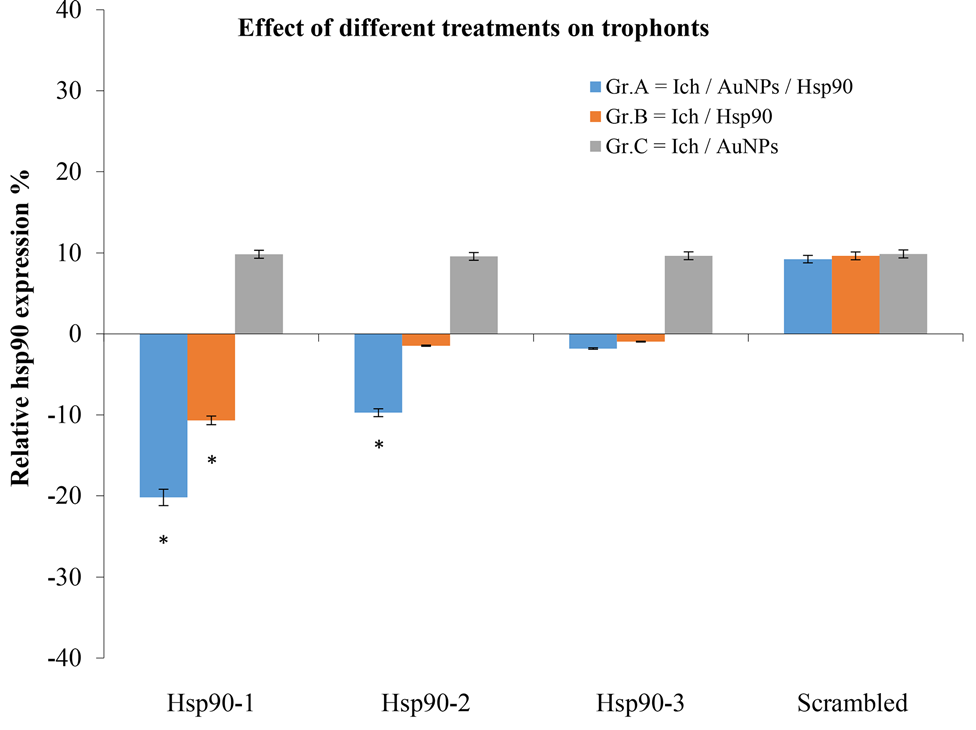
The modulation of hsp90 in trophonts after different treatments. For gene expression analysis, the relative fold change was calculated using the comparative CT method (2^−∆∆C^ T). Means for all treatments are presented normalized to the control untreated group ± SD (*n* = 3). Each sample contains ~ 25 trophonts, * = significant gene expression values (*p* < 0.05) in comparison to control

Following verification of the efficiency of the primers, the change in the transcription levels of the *hsp90* gene of *I. multifiliis* was assessed after 12 h of exposure to ASOs. qPCR showed a significant reduction (*p* < 0.05) in the expression of *hsp90* in trophonts at 12 h in three separate experiments. Different non-conjugated and gold-conjugated ASOs (hsp90-1, -2, -3, and scrambled), as well as unconjugated gold nanoparticles, were used. The ASOs hsp90-1 and hsp90-2 gold nanoparticles conjugated or non-conjugated inhibited the expression of *hsp90* in *I. multifiliis* in trophonts after transfection (Fig. 5). In contrast, hsp90-3 and scrambled ASOs displayed no inhibitory effects on trophonts.

**Fig. 6:**
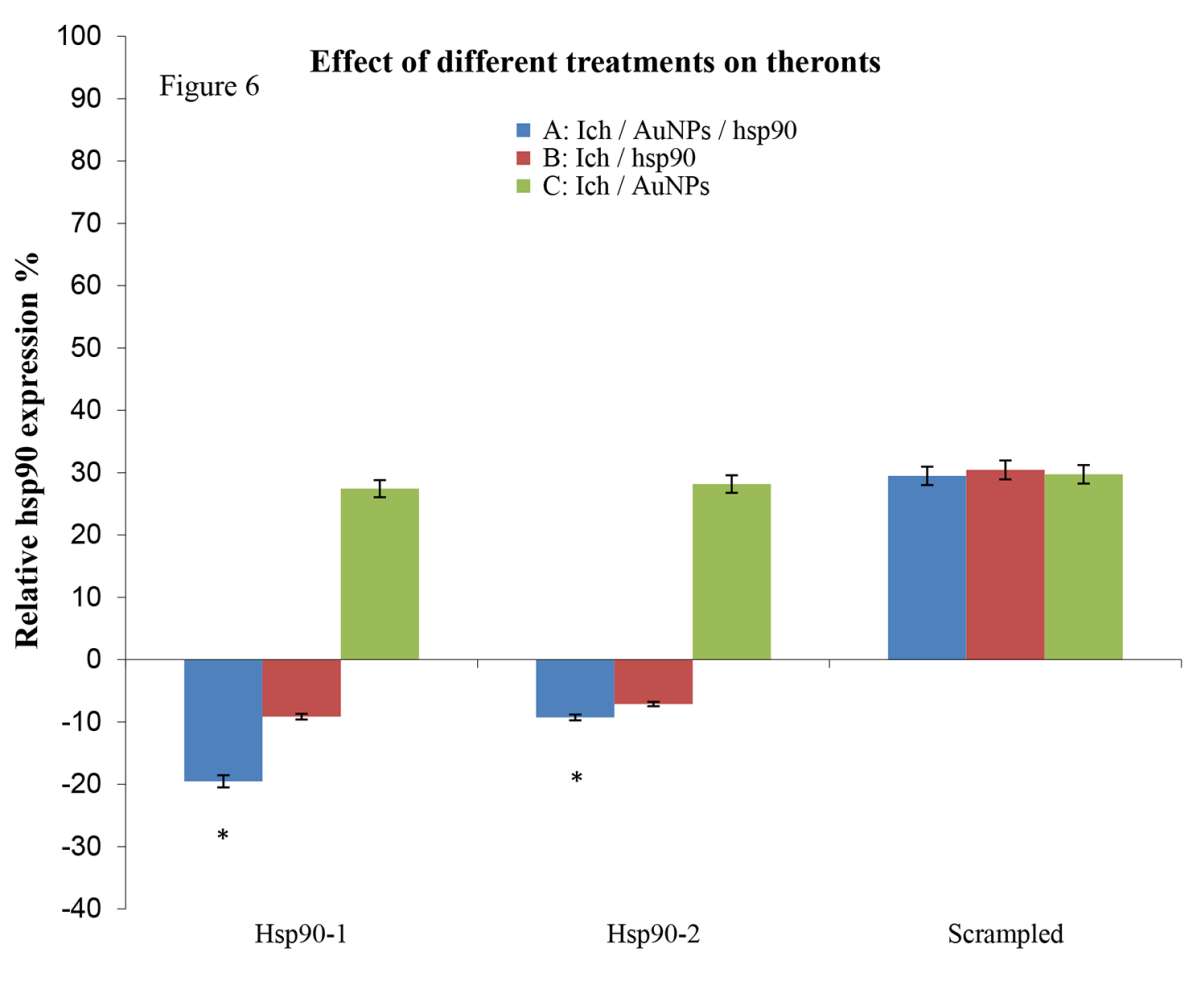
The modulation of hsp90 in theronts after different treatments. For gene expression analysis, the relative fold change was calculated using the comparative CT method (2^−∆∆C^ T). Means for all treatments are presented normalized to the control untreated group ± SD (*n* = 3). Each sample contains ~ 75 theronts, * = significant gene expression values (*p* < 0.05) in comparison to control

Consequently, the ASOs hsp90 -1 and − 2 were further analyzed to assess their inhibitory effects on theronts. Hsp90 was inhibited using gold nanoparticles conjugated to hsp90-1 and hsp90-2 (Fig. 6). However, the inhibition of hsp90 was significant (*p* **<** 0.05) in theronts when the ASO hsp90-1 was used (Fig. 6). This confirmed that ASOs-1 specifically targeted *I. multifiliis* hsp90 gene. A comparison of the effective inhibition of hsp90 using gold nanoparticles conjugated and non-conjugated to ASO hsp90-1 resulted in a significant reduction in the expression of hsp90 (*p* **<** 0.05) in both theronts and trophonts by gold nanoparticles conjugated to hsp90-1 at 12 h post-infection. In contrast, non-conjugated hsp90-1 significantly inhibited the expression of hsp90 in trophonts but not in theronts.

### Assessing the effect of different treatments on *Ichthyophthirius multifiliis*

**Fig. 7:**
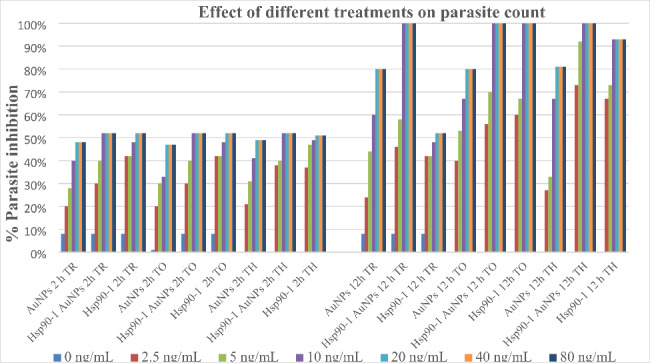
Effect of different treatments on parasite stages of *I. multifiliis*

Exposures to different concentrations of nanogold-conjugated and unconjugated hsp90-1 for 2 h resulted in ~ 50% mortality of the parasite (Fig. 7).

About half of the trophonts stopped moving and displayed slow ciliary movement. Furthermore, they stopped moving with an increase in time following exposure to 10 ng mL^− 1^ hsp90-1 nanogold-conjugated and 20 ng mL^− 1^ non-nanogold conjugated hsp90-1, respectively. The surviving trophonts exhibited asymmetric division after encystment and released theronts after 72 h. Negative controls (non-conjugated scrambled ASOs or untreated) trophonts encysted successfully and released theronts after ~ 24 h. Exposure to doses lower than 5 ng mL^− 1^ for 12 h did not significantly affect *I. multifiliis* trophonts; however, increasing the exposure time elevated the number of motionless/dead trophonts.

A 12-h exposure to 10 ng mL^− 1^ hsp90-1 nanogold-conjugated and 20 ng mL^− 1^ non-nanogold-conjugated hsp90-1 resulted in the killing of ~ 50% of incubated tomocyts. The speed of development of survivors was affected, and the subsequent release of theronts was delayed. Exposure to doses lower than 5 ng mL^− 1^ was less effective, and the tomocyts released theronts after ~ 24 h, similar to the control (scrambled ASOs or untreated)-exposed tomocyts.

The survival of theronts after exposure to different concentrations of nanogold-conjugated and non-nanogold-conjugated hsp90-1 for up to 12 h demonstrated a dose- and time-dependent response, with survival decreasing with increased concentrations of ASOs. However, increasing the concentration above 10 ng mL^− 1^ hsp90-1 gold-conjugated or higher than 20 ng mL^− 1^ unconjugated hsp90-1 did not significantly affect the mortality. With 10 ng mL^− 1^ nanogold-conjugated ASOs-1, theront survival decreased with exposure time. After 12 h, no theronts survived in this exposure group, compared with 100% survival in the scrambled control (Table 1). After 12 h, all exposed theronts were motionless. Moreover, theront survival after exposure to 20 ng non-nanogold-conjugated hsp90-1 showed a reduction over time. However, after 12 h, ~ 7% of theronts remained viable.

**Table 1:** Effects of gold nanoparticles, hsp90-1 nanogold conjugated and non nanogold conjugated hsp90-1 on *Ichthyophthirius multifiliis*. Mean of 6 wells each test (± SD) numbers **2 and 12 h post** exposure to different concentrations of treatment solutions

Solution	Solution concentrat ion (ng mL^− 1^)	Mean number of killed trophonts/well after 2 h (each well started with 25 trophonts) +/- SD	Mean % dead trophonts after 2 h	Mean number of killed tomocysts/ well after 2 h (each well started with 15) +/- SD	Mean % dead tomocysts after 2 h	Mean number of killed theronts/ well after 2 h (each well started With ~ 75) +/- SD	Mean % dead theronts after 2 h	Mean number of killed trophonts/ well after 12 h (each well started with 25 trophonts) +/- SD	Mean % dead trophontts after 12 h	Mean number of killed tomocysts/ well after 12 h (each well started with 15 tomocysts) +/- SD	Mean % dead tomocysts after 12 h	Mean number of killed theronts/well after 12 h (each well started with ~ 75 theronts) +/- SD	Mean % dead theronts after 12 h
Gold nano particles	0 2.5 5 10 20 40 80 160	2 ± 0.3 5 ± 1.7 7 ± 1.1 10 ± 1.0 12 ± 1.3 12 ± 1.9 12 ± 1.1 12 ± 1.2	8% 20% 28% 40% 48% 48% 48% 48%	0 3 ± 1.7 4 ± 1.1 5 ± 1.9 7 ± 1.2 7 ± 1.3 7 ± 1.6 7 ± 1.4	0% 20% 26.7% 33.3% 46.7% 46.7% 46.7% 46.7%	0 15 ± 1.7 23 ± 1.1 30 ± 1.9 37 ± 1.1 37 ± 1.1 37 ± 1.2 37 ± 1.3	0% 20.7% 31.3% 40.7% 49.3% 49.3% 49.3% 49.3%	2 ± 1.3 6 ± 1.7 11 ± 1.1 15 ± 1.9 20 ± 1.3 20 ± 1.1 20 ± 1.5 20 ± 1.3	8% 24% 44% 60% 80% 80% 80% 80%	0 6 ± 1.9 8 ± 1.3 10 ± 1.5 12 ± 1.1 12 ± 1.9 12 ± 1.6 12 ± 1.7	0% 40% 53.3% 66.7% 80% 80% 80% 80%	0 20 ± 1.1 25 ± 1.7 50 ± 1.9 60 ± 1.9 60 ± 1.3 60 ± 1.9 60 ± 1.2	0% 26.7% 33.3% 66.7% 80.8% 80.8% 80.8% 80.8%
Hsp90-1 nanogold conjugated	0 2.5 5 10 20 40 80 160	2 ± 1.3 8 ± 1.6 10 ± 1.1 13 ± 1.6 13 ± 1.4 13 ± 1.5 13 ± 1.3 13 ± 1.4	8% 30% 40% 52% 52% 52% 52% 52%	0 7 ± 1.6 7 ± 1.1 8 ± 1.9 8 ± 1.5 8 ± 1.8 8 ± 1.6 8 ± 1.1	0% 46.7% 46.7% 53.3% 53.3% 53.3% 53.3% 53.3%	0 28 ± 1.7 30 ± 1.9 39 ± 1.7 39 ± 1.5 39 ± 2.1 39 ± 2.2 39 ± 1.1	0% 38% 40% 52% 52% 52% 52% 52%	2 ± 1.3 13 ± 12 14 ± 1.5 25 ± 1.9 25 ± 1.5 25 ± 1.7 25 ± 1.6 25 ± 1.9	8% 46% 58% 100% 100% 100% 100% 100%	0 8 ± 1.6 10 ± 1.3 15 ± 1.6 15 ± 1.7 15 ± 1.9 15 ± 1.7 15 ± 1.4	0% 56.7% 70% 100% 100% 100% 100% 100%	0 55 ± 1.7 69 ± 1.9 75 ± 1.1 75 ± 1.3 75 ± 1.9 75 ± 1.1 75 ± 1.9	0% 73.3% 92% 100% 100% 100% 100% 100%
Hsp90-1 non nanogold conjugated	0 2.5 5 10 20 40 80 160	2 ± 1.3 11 ± 1.6 11 ± 1.8 12 ± 1.6 13 ± 1.3 13 ± 1.4 13 ± 1.7 13 ± 1.7	8% 42% 42% 48% 52% 52% 52% 52%	0 4 ± 1.1 6 ± 1.5 7 ± 1.9 8 ± 1.2 8 ± 1.3 8 ± 1.4 8 ± 1.2	0% 26.7% 40% 46.7% 53.3% 53.3% 53.3% 53.3%	0 55 ± 1.2 35 ± 1.5 37 ± 1.9 38 ± 1.3 38 ± 1.7 38 ± 1.5 38 ± 1.9	0% 36.7% 46.7% 49.3% 50.7% 50.7% 50.7% 50.7%	2 ± 1.7 15 ± 1.9 15 ± 1.1 25 ± 1.4 25 ± 1.3 25 ± 1.1 25 ± 1.3 25 ± 1.7	8% 70% 70% 100% 100% 100% 100% 100%	0 9 ± 1.3 10 ± 1.7 15 ± 1.9 15 ± 1.5 15 ± 2.1 15 ± 2.2 15 ± 1.7	0% 60% 66.7% 100% 100% 100% 100% 100%	0 50 ± 1.2 55 ± 1.5 70 ± 1.9 70 ± 1.7 70 ± 1.9 70 ± 1.5 70 ± 1.5	0 66.7% 73.3% 93.3% 93.3% 93.3% 93.3% 93.3%

### Ability of transfected theronts to subsequently infect fish

Fish co-habited with control theronts (scrambled ASO exposure) showed 100% mortality, with a mean of 90 ± 15 trophonts attached to the skin of each fish. After 12 h, no theronts survived the exposure to 10 ng mL^− 1^ nanogold-conjugated ASOs-1, and no infections were recorded in fish co-habited with this group. Fish co-habited with theronts obtained from trophonts and tomocyts exposed to 10 ng mL^− 1^ ASOs-1 were infected, with one to seven trophonts attached to each fish. These trophonts were collected; however, they failed to encyst and release theronts. Nevertheless, the infection level in this group was significantly reduced than in the control (exposed to scrambled ASOs treated theronts) group. No dead fish were recorded in the fish groups exposed to ASOs.

## Discussion

Recently, gene and genome editing tools have been increasingly used for disease management in aquaculture. We have successfully applied siRNA and Crispr/Cas9 techniques to study several fish pathogens [33–39]. In addition, gold nanoparticles non-conjugated and conjugated to different molecules have been previously used in different applications in fish medicine [40–44]. Gold nanoparticles exhibit anti-parasitic activity against several fish pathogens, including *I. multifiliis* [45, 46]. However, the antiprotozoal effect of gold nanoparticles against *I. multifiliis* was limited. Consequently, functionalization/conjugation of gold nanoparticles was thought to enhance their antiprotozoal effect [45]. Hence, we aimed to investigate the effectiveness of such modifications on the development and infectivity of *I. multifiliis*.

According to our best knowledge, due to the lack of robust methods for efficient nucleic acid delivery and genetic manipulation, functional genomic studies have not yet been performed in *I. multifiliis*. We applied antisense technology to examine the effects of targeted gene knockdown in the growth and infectivity of *I. multifiliis*. Antisense oligonucleotides and their gold nanoconjugates were used to silence the hsp90 of *I. multifiliis*.

Heat shock proteins are putative virulence factors in numerous bacteria such as *Vibrio salmonicida* and *Salmonella typhimurium*. The 66 kDa hsp of *S. typhimurium* is required for its binding to intestinal mucus [47]. The heat shock proteins (hsps) DnaK and GroEL are significantly induced in *V. salmonicida* incubated with fish skin mucus [48]. Heat shock proteins have been reported to support the survival of bacteria in their hosts [49, 50]. In addition, they have been demonstrated to be dominant antigens in the immune response to a variety of pathogens [51, 52].

In ciliates, hsp60 has been investigated during the signaling activated by cross-linking of GPI-anchored proteins (immobilization antigens) [53]. As immobilization antigen vaccine adjuvants, hsp70 provides a high level of protection in fish against *Cryptocaryon irritans* [54]. Hence, hsp90 has been suggested as a potential vaccine, and drug target and its molecular functions should be comprehensively studied. Hsp70 and hsp90 were detected in the cilia of *Tetrahymena*, with hsp90-mediated molecular interaction suggested being involved in regulating cortical patterning in *Tetrahymena* [31, 55]. Furthermore, hsp90 plays a key role in homeostasis control and the development of the protozoan parasite *Leishmania donovani*. In addition, it is involved in cell cycle control and cellular stress response [30]. Thus, we hypothesized that targeting hsp90 using antisense oligonucleotides may affect motility, development, and virulence of *I. multifiliis.*

In this study, we first developed an antisense delivery protocol in *I. multifiliis*. Second, we determined the effect of *hsp90* knockdown on the development of the parasite. Third, the infectivity of *I. multifiliis* was investigated after the silencing of hsp90. Parasite stages were separately treated with three different ASOs (hsp90-1, -2, and − 3) or scrambled ASOs as a negative control at 15 °C for 12 h. The silencing of *hsp90* expression was assessed using qPCR. We determined which ASO treatment (nanogold-conjugated and non-conjugated) was most effective in inhibiting the expression of *hsp90* in the feeding (collected trophonts) and infective (theronts) parasite stages of the *I. multifiliis*. Both nanogold-conjugated and non-conjugated ASO treatments using hsp90-1 and gold nano-conjugated hsp90-2 for 12 h at 15 °C exhibited significant *hsp90* knockdown in *I. multifiliis* trophonts. It was reported that the expression of *hsp90* was downregulated in trophonts in late passages [32]. *I. multifiliis* lose its infectivity and thereby its virulence upon a high number of passages [56]. This highlights the importance of heat shock proteins and parasite virulence.

Further, theronts were treated with ASOs hsp90-1, hsp90-2, or negative control scrambled-ASOs at 15 °C for 12 h to investigate whether the inhibition of hsp90 affected this highly infective stage. We observed that the nanogold-conjugated hsp90-1 successfully inhibited the parasite stages, including the highly infective theronts. These results were supported by the reduced number of in vitro-treated parasite stages, showing that the nanogold-conjugated hsp90-1 was highly successful.

The exposure of free-living stages of *I. multifiliis* to 10 ng mL^− 1^ gold nanoparticles, nanogold-conjugated, or non-conjugated ASOs killed 40%, 52%, and 49% of the parasites, respectively, after 2 h of exposure. Trophonts surviving the exposures successfully transformed into tomocysts; however, these showed asymmetric cell division and/or delayed (72 h compared with 24 h for control) development time to release theronts, as reported by Saleh et al. [45]. Although lower concentrations affected the parasite metabolism and delayed theronts release, these were less effective in killing encysted tomocysts. After 12 h, we observed significantly higher mortalities (100%) compared to (~ 34%) theronts after exposure to ≥ 10 ng mL^− 1^ gold nanoparticles than nanogold-conjugated ASO treatments. This was attributed to the enhanced specificity of designed hsp90-1 ASOs, which likely affected the viability of theronts. In vitro assays revealed that non-nanogold-conjugated hsp90-1 reduced the number of theronts over time; however, those that survived the exposure could still infect fish.

Previous work has shown gold nanoparticles to have limited activity against *I. multifiliis* [45]. However, no previous assessment has been reported in the literature using ASOs or ASO-conjugated gold nanoparticles on different free-living developmental stages of *I. multifiliis*, which are considered major factors for the occurrence and distribution of white spot disease. Further, specific-pathogen-free common carp in water were inhabited by hsp90-1-treated *I. multifiliis*. The carp did not develop white spots on the skin or gills. Altogether, these results demonstrate a novel ASO-based functional study in *I. multifiliis*.

Taken together, nanogold-conjugated ASOs used in this study were effective against all *I. multifiliis* free-living stages. The gold nanoparticle-conjugated antisense approach introduced in this study provides insights into the functions of hsp90 in *I. multifiliis*. Their action likely affects the movement, virulence, and development of *I. multifiliis*. However, the precise mechanism underlying the effect of nanogold-conjugated hsp90 ASOs needs to be further investigated. The results obtained from this study contribute to our understanding of parasite pathogenicity and could help us identify additional drug targets for effective therapeutics and disease management. Furthermore, this study provides a supportive research approach and establishes useful tools to investigate the gene function in *I. multifiliis*, paving the way for further genomic studies on this devastating protozoan parasite.

## Materials and methods

### Ethics Statement

All experiments were approved by the Animal Experimentation Ethics Committee of Vienna University of Veterinary medicine (BMWFW-68.205/0051-WF/V/3b/2016). All experiments were executed in agreement with relevant guidelines and regulations to minimize suffering of the fish. The current study was performed at the experimental facility of Department for Farm Animals and Veterinary Public Health, Clinical Division of Fish Medicine, University of Veterinary Medicine, Austria. Procedures for animal care and management were conducted according to the guidelines of the European institutional ethics and animal welfare after the approval from the Ministry of Science, Austria according to § 26ff of the Austrian laws for care and use of experimental animals (BMWFW, GZ: 2020 − 0.001.578).

### Laboratory propagation of *Ichthyophthirius multifiliis*

The laboratory life cycle of *I. multifiliis* was initiated by co-habitation of naïve rainbow trout (*Oncorhynchus mykiss*) with naturally infected giant gourami (*Osphronemus goramy*), according to Saleh et al. [45]. They were kept in constantly flowing tap water at 16 ± 2 °C. Heavily infected fish were euthanized in 0.05% (w/v) tricaine methanesulfonate (MS-222, Sigma-Aldrich, Vienna, Austria), following which the skin mucus layer was scraped into Petri dishes containing 10 mL of water at 16 °C. When the trophonts escaped from the mucus, they were collected with a pipette and transferred to clean water in Petri dishes. After incubation for 18 h at 14 °C, the trophonts developed to tomocyts and subsequently were encysted. These were transferred to clean water in Petri dishes. Between 43 and 48 h, after the initial isolation of trophonts, the theronts were released from the tomocyts. The time required for the formation of tomocyts and the release of theronts from a trophont can be extended by keeping the parasite at 10 °C to provide sufficient time for accomplishing and monitoring the antisense delivery on different development stages.

The life cycle of the parasite was divided into three distinct stages. The trophont resides and feeds on the epidermis of the host, where it can attain a diameter of up to approximately 1 mm. The mature trophont escapes from the epidermis to the surrounding water, where certain parasites settle and develop into tomocyts. Inside the cysts, the parasite repeatedly divides to produce daughter cells (tomites). The number of tomites resulting from one tomocyt varies between 50 and several thousand [4, 46]. These stages escape the cyst as theronts, ready to infect the fish epithelium. The stages of the life cycle are highly dependent on the temperature. Thus, the time required for the development and release of theronts from a trophont is as long as 9 days at 5 °C; however, it is drastically reduced to 18 h at 25 °C [4, 46].

### Collection of parasite stages

Following experiments performed previously, various stages of the parasite were collected [46]. Briefly, trophonts were selected and collected in batches of 25 in Petri dishes filled with 10 mL of filtered fresh water and either used directly or incubated at 15 ± 1 °C, until they reached either the tomocyt stage (minimum 8 cells) after ~ 6 to 12 h or the theront stage (after ~ 23–30 h). To obtain tomocyts, Petri dishes with trophonts were incubated for only 16 h at 15 °C so that the development of theronts was not achieved.

Theronts were released by placing 25 trophonts into Petri dishes with 10 mL of water and incubating at 15 °C for 24 h [57]. To determine the number of theronts produced, 10 × 20 µL of subsamples were placed on slides fixed with 5 µL of the Roti Histofix (Carl Roth) and counted under a microscope. The mean count was used to assess the total number of theronts produced. Active parasites were considered viable, whereas damaged and dead parasites were motionless. A dual-fluorescent staining technique using propidium iodide (PI) and fluorescein diacetate (FDA) was occasionally used to differentiate between viable and damaged parasites by fluorescent microscopy [46].

### Design, selection, and synthesis of *Ichthyophthirius multifiliis*-specific antisense oligonucleotides

We targeted the *I. multifiliis* hsp90 mRNA with antisense oligonucleotides (ASOs) to silence or knock down its expression and subsequently investigate the development, virulence, and infectivity of the parasite. We designed three complementary ASOs, namely, hsp90-1, -2, and − 3 using the antisense design program (IDT Integrated DNA Technologies, USA); which specifically target and bind to distinct regions of *I. multifiliis* hsp90 mRNA (Table 2). The oligonucleotides were analyzed for probable RNA secondary structures and target accessibility by Sfold and mfold. The specificity of ASOs was confirmed by BLAST search against the fish genome, and related ciliate and fish pathogen sequences available in the GenBank. The ASOs and scrambled control sequences were commercially synthesized and used for transfection experiments. Thiol (for efficient conjugation with gold nanoparticles and transfection)-modified ASOs were labeled with a fluorescent dye to monitor their delivery into *I. multifiliis* by fluorescence microscopy.

**Table 2:** Sequences of ASOs completely complementary to hsp90 (GenBank accession number: XM_004035695.1) of *Ichthyophthirius multifiliis*

Name Sequence (5’-3’) Position of the ASOs
Hsp90-1: 5’- Cy5-AAAUAACUUCGACUCUCUCA-(C3 S-S)-3’ 482–501
Hsp90-2: 5’- Cy5-UGUUCUAUUCUUCGAAUUCG-(C3 S-S)-3’ 866–885
Hsp90-3: 5’- Cy5-AACACAAGGAGAUUCAUCUA-(C3 S-S)-3’ 1786–1805
Hsp90-C: 5’- Cy5-CCAACCUGGGCAUUGCAGAA-(C3 S-S)-3’ -

### Delivery of antisense oligonucleotides to *Ichthyophitirius multifiilis*

One of the factors critical to successful antisense experiments is the efficiency with which ASOs are transfected into the cells of interest. We compared diverse transfection conditions and different methods for introducing ASOs into the parasite (e.g., soaking and gold nanoparticles). The hsp90-ASOs were used to transfect trophonts, tomocyts, and theronts.

### Soaking

Trophonts, tomocyts, and theronts were separately treated with ASOs (hsp90 -1, -2, -3, and scrambled) or left untreated. The ASOs were added to the parasite stages with or without a commercial transfection agent (Lipofectamine 2000, Invitrogen, USA) to assess the efficiency of antisense uptake into the parasite. Concentrations of ASOs ranged from 1 to 3 mM (final concentration in the medium) and different ratios of ASOs/Lipofectamine 2000 (mM ASOs/mL Lipofectamine 2000 = 1:1; 1:2; 1:3) were checked.

Following the transfection, to monitor the effect of different treatments on parasite movement and development, trophonts, tomocyts, and theronts were incubated for 24 h. For RNA extraction and expression analysis of the hsp90 by quantitative real-time polymerase chain reaction (PCR), transfected trophonts and theronts were collected separately after 12 h and preserved in RNAlater.

### Gold nanoparticle synthesis

Gold nanoparticles were surface-functionalized with hsp90-thiol-modified ASOs according to the method described by Rosi et al. [22]. Briefly, we synthesized citrate-stabilized gold nanoparticles by reducing tetrachloroauric acid (HAuCl_4_) with sodium citrate [45, 46]. An aqueous solution of HAuCl_4_.3H_2_O was boiled under reflux with stirring. After rapid addition of 10 mL of 1% trisodium citrate, the color of the solution changed from yellow to deep red. After an additional 15 min of reflux, the solution was allowed to cool to room temperature, before being filtered through a 0.45 μm acetate filter and subsequently stored at 4 °C.

### Characterization of nanoparticles

The formation of gold nanoparticles was confirmed by ultraviolet–visible spectral analysis. The absorbance spectra were recorded using NanoDrop 2000. Deionized water was used as blank. All measurements were performed at room temperature on 3 different days. The morphology of the synthesized gold nanoparticles was analysed using transmission electron microscopy (TEM; EM 900, Zeiss, Oberkochen, Germany). The Image SP Viewer software was used to estimate their mean size from 100 arbitrarily tested nanoparticles. We used a Zetasizer Nano ZS (Malvern.com) to measure the size distribution of nanoparticles based on dynamic light scattering (DLS). Triplicate measurements were performed at room temperature.

### Preparation of oligonucleotide-modified gold nanoparticles

Gold nanoparticle probes (ASNPs) were synthesized according to Rosi et al., with some modifications [22]. Briefly, the ASNPs were prepared by derivatizing an aqueous 13 nm diameter after confirmation and characterization as described above AuNP solution (10 nM) with thiol-modified ASO (2 µM). After standing for 16 h, the solution was brought to 0.1 M NaCl, 10 mM phosphate buffer (pH 7), and allowed to stand for 40 h, followed by centrifugation for at least 25 min at 14,000 rpm to remove excess reagents. Subsequent to removal of the supernatant, the precipitate was washed with 5 mL of a stock of 0.1 M NaCl, 10 mM phosphate buffer (pH 7) solution, recentrifuged, and redispersed in 5 mL of a 0.3 M NaCl, 10 mM phosphate buffer (pH 7), 0.01% azide solution.

### Transfection of *I. multifiliis*

The parasite stages were transfected separately with each designed ASO (target-specific hsp90 -1, -2, -3, and scrambled) or left untreated using the optimized delivery method. To monitor the successful transfection of *I. multifiliis*, we used 5’ Cy5 fluorescently labeled scrambled oligonucleotides and observed their uptake under a fluorescence microscope.

RNA extraction, cDNA synthesis, and gene expression analysis by qRT-PCRTrophonts and theronts were treated with non-conjugated, gold-conjugated 10 nM ASOs (hsp90-1, -2 and − 3, and scrambled), or unconjugated gold nanoparticles. Trophonts and theronts were collected in RNAlater for RNA extraction and quantitative PCR (qPCR) analysis. The total RNA was isolated from antisense-treated and non-treated parasite stages using the RNeasy Mini Kit (Qiagen, Hilden, Austria) according to the manufacturer’s instructions. The integrity and quality of the extracted RNA was evaluated by adding Invitrogen™ NorthernMax™ formaldehyde loading dye and using 1% agarose gel electrophoresis. In addition, the UV absorption of the samples was measured at 260 nm. An on-column DNase digestion step was included, and RNA samples were quantified using a NanoDrop 2000 spectrophotometer (Thermo Scientific, Vienna, Austria). Using an iScript cDNA Synthesis Kit (Bio-Rad, Vienna, Austria), cDNA was synthesized with 1 µg of total RNA according to the user’s manual.

The efficiency of gene knockdown was evaluated by quantitative real-time PCR. The primers 5′-AAC CAA CAC CAA ATT GCC CA-3′and 5′-CTG ATG CCG AAA GTG CCA GA-3′for *I. multifiliis* hsp90 (GenBank accession number: XM 004035695.1) were checked and optimized to assess the gene expression by qPCR amplifying a segment of 138 bp. The 18 S rRNA (GenBank accession number: U17354.1) primers were used as a reference gene as reported by Abernathy et al. [32]. The forward primer was 5- GTGACAAGAAATAGCAAGCC-3 and the reverse primer was 5- CCCAGCTAAATAGGCAGAAG-3. These primers amplified a segment of 193 bp. The primer sets were tested to determine the optimal annealing temperature and primer concentration. qRT-PCR was performed according to the method described by Abernathy et al. [32] with some modifications.

Briefly, qRT-PCR was executed by means of a Bio-Rad iCycler following the manufacturer’s instructions in a total volume of 20 µL: 5 µl of 1:2-fold diluted cDNA, 10 pmol of each primer, 2× SsoAdvanced Universal SYBR Green Supermix (Bio-Rad), and diethylpyrocarbonate (DEPC)-treated sterile distilled water. Each qRT-PCR was performed in triplicate. After 2 min of denaturation at 95 °C, 50 cycles were performed at 95 °C for 5 s, 56 °C for 10 s, and 72 °C for 20 s. For detecting non-specific binding, a melting-point curve was evaluated, at temperature from 53 °C with a rise of 0.5 °C at every single 10 s up to 95 °C. Standard curves were obtained for hsp90 and 18 S rRNA genes to verify the efficiency of the primers.

Assessing the effect of different treatments on *Ichthyophthirius multifiliis*.

### Effect of different treatments on trophonts

Approximately, 25 trophonts in 500 µL of the molecular biology reagent water were placed in each well of a 24-well tissue culture plate. Different concentrations (0, 2.5, 5 10, 20, 40, 80, or 160 ng mL^− 1^) of hsp90-1 nanogold-conjugated, non-nanogold conjugated, and gold nanoparticles were added separately in triplicates. Control wells included scrambled or molecular biology reagent water. The efficacy of the inhibition was assessed by counting the number of trophonts at 15 and 30 min, 1 h, 2 h, 4 h, 6 h, 12 h, 18 h, and 24 h until the trophonts were either dead or theronts were released. Trophonts were classified as active (survival) or motionless (dead) using a microscope (40×), as previously described [45]. The numbers of tomocyts and released theronts were assessed at 6 and 24 h post-transfection, respectively.

### Effects of different treatments on tomocyts

Approximately, 15 tomocyts in 500 µL of the molecular biology reagent water (Sigma-Aldrich, Vienna, Austria) were placed in each well of a 24-well tissue culture plate. Fewer number of tomocyts (*n* = 15) were used because not all trophonts were encysted successfully. Afterward, 500 µL aliquotes of hsp90-1 nanogold-conjugated, non-nanogold conjugated, and gold nanoparticles were separately added in triplicates. Tomocyts were categorized as active (survival) or motionless (dead) as above. The numbers of released theronts were determined 24 h post-transfection.

### Effect of different treatments on theronts

In triplicates, wells of 24-well plates were filled with 500 µL of the theront suspension with ~ 75 theronts counted as defined above. Next, 500 µL of hsp90-1 nanogold-conjugated, non-nanogold conjugated, and gold nanoparticles or control was separately added to the wells in triplicates. The number of theronts surviving in each well was determined at 30 min, 1 h, 2 h, 4 h, 8 h, 12 h, 18 h, and 24 h post-transfection. Each triplicate was obtained from the same group of parasites to decrease the possible different survival rates between diverse cohorts.

### Ability of transfected theronts to subsequently infect fish

*Ichthyophthirius multifiliis* was maintained in our laboratory using rainbow trout as the host. Rainbow trout were used as a well established model in our lab for the propagation of the parasite to get constant and enough quantities of the parasite stages for the optimization and accomplishment of the in vitro studies. In our previous study, the hsp90 protein was identified in the *I. multifiliis* infected carp mucus [27]. Hence, common carp were used for the in vivo experiment. *I. multifiliis-*transfected theronts, theronts from the transfected trophont or tomocyts were used to infect common carp via bathing to evaluate if hsp90 inhibition affected the virulence of the parasite and disease development in the host. The results were evaluated in comparison with control infected fish.

To determine whether ASO-treated theronts had the ability to infect fish, Petri dishes in triplicates were prepared, each containing 12 mL of theront suspension (~ 75 theronts mL^− 1^) drawn from one pool of treated trophonts, tomocyts, or theronts. The number of live versus dead theronts in three separate 1 mL aliquots taken from each Petri dish was determined. The remaining 47 mL was added to separate tanks of common carp to determine if surviving theronts could infect fish.

Four sets of triplicate 100 L tanks were maintained at a constant temperature at 15 °C for the infection trial. Each tank contained 20 (~ 5 g) *C. carpio*. In each tank, the common carp were exposed to the appropriate batches of theronts for 3 h under static conditions in the dark with aeration. Fish were maintained at 15 °C on a 2% body weight d^− 1^ ratio of commercial feed diet (Garant Aqua, Pöchlarn, Austria). Fish were then euthanized using an overdose of 0.05% (w/v) MS-222 (Sigma-Aldrich, Austria). The total numbers of trophonts on the fins, gills, and entire body surface were recorded.

### Ability of trophonts collected from fish after infection with theronts surviving hsp90 inhibition to subsequently encyst into tomocyts and release theronts

Trophonts were collected from infected fish 10 days post-exposure (from above) and placed onto Petri dishes containing 10 mL of water at 15 °C. Their ability to encyst into tomocyts and release theronts was observed at 6 and 24 h, respectively.

### Statistical analysis

Gene expression levels were statistically assessed. Differences between all groups were evaluated using *t*-tests with Bonferroni’s α-correction. Relative fold change was normalized to 18 S rRNA and subsequently expressed as a fold change relative to expression levels of control untreated group. For gene expression analysis, the relative fold change was calculated using the comparative CT method (2^−∆∆C^ T). Means for all treatments are presented normalized to the control untreated group ± SD (*n* = 3). For all statistical tests, a *p-*value **<** 0.05 was considered significant.

The inhibition rate of *I. multifiliis* was calculated as percent inhibition = 100−[(mean number of viable parasites counted in exposed wells/mean number of parasites counted in non-exposed wells) × 100]. The differences between nanoparticles exposed and non-exposed parasites were analyzed using *t-*tests with Bonferroni’s α-correction. For all statistical tests, a *p-*value **<** 0.05 was considered significant. Statistical analyses were conducted using the SPSS V.20 software.

## Data Availability

The datasets generated during and/or analyzed during the current study are presented in the manuscript.

## Abbreviations

ASOs: Antisense oligonucleotides
AuNPs: Gold nanoparticles
DLS: Dynamic light scattering
ASNPs: Oligonucleotide-AuNP conjugates
TEM: Transmission electron microscopy
UV–Vis: Ultraviolet visible spectrophotometry
Hsps: Heat shock proteins
hsp90: Heat shock protein 90
TH: Theronts
TO: Tomonts
TR: Trophonts.
